# Arthritic Microenvironment Actuated Nanomotors for Active Rheumatoid Arthritis Therapy

**DOI:** 10.1002/advs.202204881

**Published:** 2022-11-14

**Authors:** Cong Xu, Yuejun Jiang, Hong Wang, Yuxin Zhang, Yicheng Ye, Hanfeng Qin, Junbin Gao, Qing Dan, Lingli Du, Lu Liu, Fei Peng, Yingjia Li, Yingfeng Tu

**Affiliations:** ^1^ Department of Medicine Ultrasonics Nanfang Hospital Southern Medical University Guangzhou 510515 China; ^2^ School of Pharmaceutical Sciences Guangdong Provincial Key Laboratory of New Drug Screening Southern Medical University Guangzhou 510515 China; ^3^ Department of Ultrasound First Affiliated Hospital of Guangzhou Medical University Guangzhou 510120 China; ^4^ School of Materials Science and Engineering Sun Yat‐Sen University Guangzhou 510275 China

**Keywords:** macrophage regulation, nanomotor, oxygen generation, rheumatoid arthritis, ultrasound

## Abstract

Increasing O_2_ demand and excessive ROS production are the main features of arthritic microenvironment in rheumatoid arthritis (RA) joints and further play pivotal roles in inflammation exacerbation. In this work, a system of in situ regulation of arthritic microenvironment based on nanomotor strategy is proposed for active RA therapy. The synthesized MnO_2_‐motors enable catalytic regulation of RA microenvironment by consuming the overproduced H_2_O_2_ and generating O_2_ synergistically. The generated O_2_ under H_2_O_2_‐rich conditions functions as inflammation detector, propellant for enhanced diffusion, as well as ameliorator for the hypoxic synovial microenvironment. Owing to O_2_ generation and inflammation scavenging, the MnO_2_‐motors block the re‐polarization of pro‐inflammatory macrophages, which results in significantly decreased secretion of multiple pro‐inflammatory cytokines both in vitro and in vivo. In addition, intra‐articular administration of MnO_2_‐motors to collagen‐induced arthritis rats (CIA rats) effectively alleviates hypoxia, synovial inflammation, bone erosion, and cartilage degradation in joints. Therefore, the proposed arthritic regulation strategy shows great potential to seamlessly integrate basic research of RA with clinical translation.

## Graphical abstract

In this work, a system of in situ regulation of arthritic microenvironment based on nanomotor strategy is proposed for active RA therapy. The synthesized MnO_2_‐motors enable catalytic regulation of RA microenvironment by consuming the overproduced H_2_O_2_ and generating O_2_ synergistically. This arthritic microenvironment powered nanomotor system simultaneously integrates hypoxia relief and ROS scavenging for macrophages phenotypic regulation.

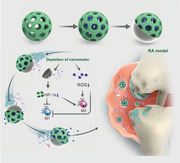

## 1 Introduction

Rheumatoid arthritis (RA) is a chronic inflammatory disorder involving multiple inflammatory cells, and RA can result in lifelong joint disabilities.^[^ ^1^ ^]^ The joint synovium of RA is normally infiltrated by numerous inflammatory cells, especially macrophages, which play a pivotal role in RA development.^[^ ^2^ ^]^ The predominant phenotype of macrophages in RA joints is pro‐inflammatory M1 macrophages, featured by an overexpression of inflammatory cytokines such as tissue necrosis factor alpha (TNF‐*α*), interleukin 1*β* (IL‐1*β*), and interleukin 6 (IL‐6).^[^ ^3^ ^]^ This is while anti‐inflammatory M2 macrophages, to the contrary, are capable of secreting anti‐inflammatory cytokines for the relief of inflammation.^[^ ^4^ ^]^ As a result of synovial tissue proliferation outpacing angiogenesis, increasing cellular demand for oxygen during the inflammatory process in RA joint further induces the hypoxic microenvironment and up‐regulates hypoxia‐inducible factor (HIF‐1*α*) expression.^[^ ^5^ ^]^ It had been reported that the overexpression of HIF‐1*α* in RA was able to affect the balance of macrophages polarization from M2 into M1 phenotypes.^[^ ^5^, ^6^ ^]^ Meanwhile, the existing reactive oxygen species (ROS) are also closely related to the inflammation progress in arthritis, showing strong M1 activation performance through specific M1 signaling pathway (NF‐kB).^[^ ^7^ ^]^ Therefore, to develop a new strategy for HIF‐1*α* inhibition and ROS scavenging to block M1 phenotypic polarization is of great significance for RA therapy.

Hydrogen peroxide (H_2_O_2_) is known as one of the main ROS that arise through oxidative metabolism in inflammatory diseases.^[^ ^8^ ^]^ However, if not consumed timely, H_2_O_2_ with high concentration may result in severe tissue damage and chronic inflammation.^[^ ^9^ ^]^ Thus, enzyme‐like nanoparticles (NPs) for H_2_O_2_ decomposition have been investigated to avoid H_2_O_2_‐mediated deleterious effects.^[^ ^10^ ^]^ Among various catalytic NPs, MnO_2_ NPs with high biocompatibility have been developed for sustained oxygen generation during H_2_O_2_ decomposition.^[^ ^3^, ^11^ ^]^ The continuously generated oxygen is possible to relieve the microenvironmental hypoxia for both anti‐cancer and anti‐inflammation treatment. Mn^2+^ from degradation is also nontoxic and can be excreted by hepatic and renal systems.^[^ ^12^ ^]^ However, it is difficult to achieve desirable efficacy because of the limited uptake of these MnO_2_‐based nanoagents with passive diffusion^[^ ^13^ ^]^ and the reduction in oxygen supply due to the fact that hydroxyl radicals are generated as intermediates from the Fenton reaction.^[^ ^14^ ^]^ Ceria NPs) have been recently developed as a multi‐antioxidant for ROS scavenging.^[^ ^15^ ^]^ However, a high concentration of ceria NPs can cause safety concerns associated with their cytotoxicity concerns.^[^ ^16^ ^]^ Therefore, the biomedical applications of these NPs would be vastly widened by reducing the dosage and endowing them excellent diffusion capability as well as efficient elimination of the toxic reaction products simultaneously.

**Figure 1:**
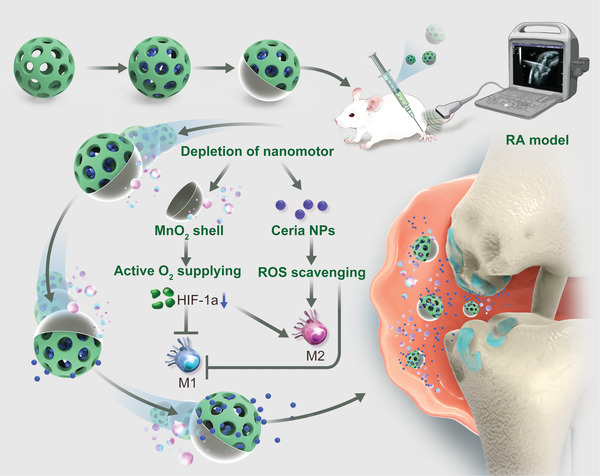
Schematic illustration of in vivo therapeutic mechanism using MnO_2_‐motors for hypoxic inflamed joints through active O_2_ generation and ROS scavenging. The arthritic microenvironment powered nanomotor system simultaneously integrates hypoxia relief and ROS scavenging for macrophages phenotypic regulation.

Inspired by mobile behavior of organisms, micro/nanomotors that can convert surrounding energies (chemical or physical energies) into mechanical motion have drawn extensive attention in the past decades.^[^ ^17^ ^]^ By decomposing H_2_O_2_, water and so on, chemically driven motors with active motion capability have shown enormous potential in biomedical applications ranging from diagnostic imaging^[^ ^17^, ^18^ ^]^ and targeted drug delivery^[^ ^19^ ^]^ to minimally invasive surgery.^[^ ^17^, ^20^ ^]^ As widely‐used chemical‐fuel powered motors, micro/nanomotors based on H_2_O_2_ decomposition present active propulsion in a H_2_O_2_‐dependent behavior.^[^ ^21^ ^]^ However, addition of H_2_O_2_ into biological environment is not feasible because of its high toxicity. Therefore, adopting the naturally‐presented H_2_O_2_ within microenvironment as chemical fuel to drive biomedical nanomotors shows superiority for further in vivo application. Meanwhile, the generated oxygen under body fluids can be used as contrast agent for ultrasonic imaging due to the change of acoustic impedance.^[^ ^22^ ^]^ Herein, we proposed a H_2_O_2_‐actuated MnO_2_ nanomotor system (MnO_2_‐motor) with hypoxia relief and ROS scavenging for catalytic regulation of synovial microenvironment in RA. By decomposing the local H_2_O_2_ from RA microenvironment, the resulting MnO_2_‐motor possessed enhanced diffusion along with continuous oxygen generation. Anchored with ceria NPs, MnO_2_‐motor alleviated hypoxia as well as scavenged ROS in pro‐inflammatory macrophages, inhibiting M1 phenotypic polarization to postpone disease progression (**Figure** **1**). Furthermore, with the help of ultrasound, RA progression assessment toward H_2_O_2_‐sensitive oxygen generation of MnO_2_‐motor was investigated in a rat model with collagen‐induced arthritis (CIA).

## 2 Results and Discussion

### 2.1 Fabrication and Characterization of MnO_2_‐Motors

**Figure 2:**
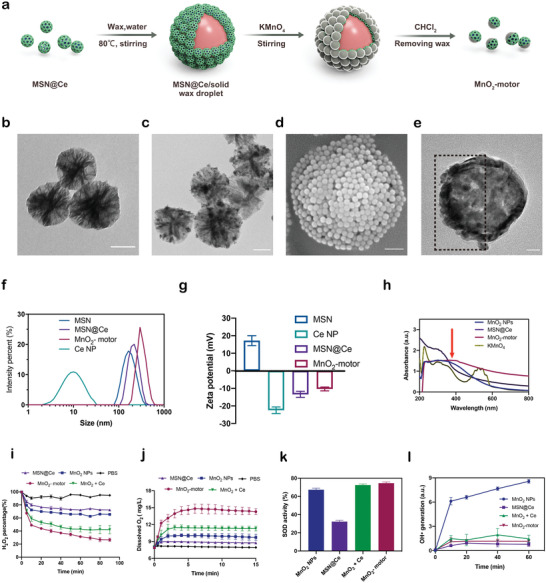
Fabrication and characterization of MnO_2_‐motors. a) Schematic illustration of the fabrication of MnO_2_‐motors. b) TEM image of MSN. Scale bar = 100 nm. c) TEM image of MSN@Ce. Scale bar = 100 nm. d) SEM image of MSN@Ce /solid wax droplet. Scale bar = 1 µm. e) TEM image of MnO_2_‐motors. Scale bar = 50 nm. f) Size distribution of MSNs, ceria NPs, MSN@Ce, and MnO_2_‐motors. g) Zeta potential of MSNs, ceria NPs, MSN@Ce, and MnO_2_‐motors. h) UV–vis spectra of KMnO_4_, MnO_2_ NPs, MSN@Ce, and MnO_2_‐motors. i) H_2_O_2_ degradation curves in the presence of MnO_2_ NPs, MSN@Ce, MnO_2_ + Ce, and MnO_2_‐motors under 2.5 mm H_2_O_2_ solution (*n* = 4; mean ± SD). j) O_2_ generation curves in 10 mm H_2_O_2_ solution under physiological conditions (*n* = 4; mean ± SD). k) SOD activity of MnO_2_ NPs, MSN@Ce, MnO_2_ + Ce, and MnO_2_‐motors (*n* = 4; mean ± SD). l) Time‐dependent hydroxyl radical generation of MnO_2_ NPs, MSN@Ce, MnO_2_ + Ce, and MnO_2_‐motors in the presence of 1 mm H_2_O_2_ (*n* = 4; mean ± SD).

The fabrication of MnO_2_‐motors mainly consists of two steps: the synthesis of ceria NPs loaded large pore mesoporous silica NPs (MSN@Ce) and the asymmetric MnO_2_ coating on MSN@Ce using pickering emulsion approach (**Figure** **2**a). In the first step, MSNs were synthesized according to the previous report^[^ ^23^ ^]^ with several modifications, followed by ceria NPs loading through electrostatic interactions. The structure of ceria NPs was confirmed by transmission electron microscopy (TEM) and X‐ray powder diffraction (Figure S1, Supporting Information). To confirm the conjugation of ceria NPs to MSNs, the morphology was then characterized by TEM (Figure 2b,c). Compared with free MSNs (Figure 2b), ceria NPs were successfully anchored in the pores of MSNs, as shown in Figure 2c. The hydrodynamic size of the resulting MSN@Ce was increased from 282.2 ± 5.5 nm (MSNs) to 298 ± 4.7 nm, indicating successful ceria NPs loading from another point of view. Next, MSN@Ce was decorated onto the wax droplets via pickering emulsion. Pickering emulsion is a promising approach to synthesize Janus particles with high yields. It consists in preparing an emulsion stabilized by colloids to be partially modified: as one side of the particles is masked by solid wax at room temperature, the other side can then be chemically modified in a second step.^[^ ^24^ ^]^ A monolayer of MSN@Ce was anchored regularly onto the wax droplet as observed by SEM (Figure 2d). After addition of potassium permanganate (KMnO_4_) solution, a MnO_2_ shell was then successfully grown onto the surface of MSN@Ce/solid wax droplets. Subsequently, MnO_2_‐motors were finally obtained by dissolving the wax with chloromethane. The asymmetrical MnO_2_‐motors were clearly observed under TEM (Figure 2e), with an average diameter of 342 ± 7.8 nm, a bit larger than that of MSN@Ce (Figure 2f). According to Figure S2, Supporting Information, manganese, oxygen, and silica on the surface of MnO_2_‐motor were observed through energy‐dispersive X‐ray spectroscopy (EDX), further demonstrating the successful formation of MnO_2_ layer. As shown in Figure 2g, the surface zeta potential of the resulting MnO_2_ nanomotors was increased (−9.8 mV) compared to that of MSN@Ce (−13.6 mV) after coating MnO_2_ shell. UV–vis spectrum of MnO_2_‐motors was also measured and MnO_2_‐motors displayed a broad absorption peak around 380 nm (Figure 2h), which was consistent with MnO_2_ NPs.

Next, the catalytic capability of MnO_2_‐motors with those of MnO_2_ NPs and MSN@Ce was compared to investigate the synergistic effect of H_2_O_2_ decomposition and O_2_ generation. In a time‐dependent H_2_O_2_ assay under PBS, 34.5% and 27.5% of H_2_O_2_ was decomposed after 1.5 h by MnO_2_ NPs and MSN@Ce, respectively (Figure 2i). Interestingly, the majority of H_2_O_2_ up to 61.9% was decomposed by MnO_2_‐motors (containing the same amounts of ceria with MSN@Ce and manganese with MnO_2_ NPs according to ICP‐MS analysis) after only 30 min, demonstrating dramatically improved catalytic effect of our MnO_2_‐motors. According to Figure 2j, MnO_2_‐motors displayed a quick and sustained O_2_ releasing behavior through markedly increased dissolved oxygen value compared with that of MnO_2_ NPs and MSN@Ce. In addition, the physical mixture of MnO_2_ NPs and MSN@Ce (MnO_2_ + Ce) rarely showed obvious improved effect on H_2_O_2_ decomposition and O_2_ generation. The outstanding H_2_O_2_ decomposition and O_2_ generation indicates enhanced diffusion of our MnO_2_‐motors owing to the typical Janus structure in the presence of H_2_O_2_ fuel.

More importantly, ceria NPs have been reported to possess multiple ROS scavenging capability by removing ^•^OH and O^2•−^ via redox reactions or superoxide dismutase (SOD) mimetics,^[^ ^15^, ^25^ ^]^ while MnO_2_ NPs are well known to generate ^•^OH through Fenton‐like activity.^[^ ^14^, ^26^ ^]^ To understand this particular effect on our MnO_2_‐motors, the activity of SOD and hydroxyl radicals were further explored, which are intermediates in the intracellular H_2_O_2_ metabolism pathway. As shown in Figure 2k, all NPs possessed SOD activity, but no apparent synergistic effect on SOD activity was observed for the group of MnO_2_ + Ce. The hydroxyl radical generation was then evaluated after adding H_2_O_2_. The results confirmed that MnO_2_ NPs generate hydroxyl radicals as an intermediate of the Fenton reaction (Figure 2l). Surprisingly, MnO_2_‐motors and MnO_2_ + Ce did not produce hydroxyl radicals, indicating that ceria NPs scavenged these radicals produced by MnO_2_ NPs. In addition to the enhanced diffusion, the synergistic effect of MnO_2_‐motors on efficient H_2_O_2_ decomposition and O_2_ generation could be attributed to the conversion of hydroxyl radicals to O_2_ molecules by ceria NPs during the Fenton reaction of MnO_2_ NPs. This highly integrated nanomotor system provides a novel platform for synergistic O_2_ generation and new possibility for inflammatory disease treatment.

### 2.2 Enhanced Diffusion of MnO_2_‐Motors

**Figure 3:**
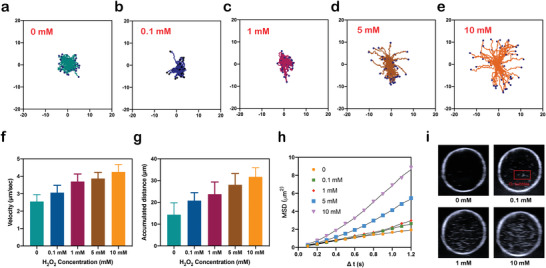
Motion of MnO_2_‐motors. a–e) Enhanced diffusion of MnO_2_‐motors in the presence of different H_2_O_2_ concentrations. f) Velocity of MnO_2_‐motors. g) Accumulated distance of MnO_2_‐motors. h) MSD curve of MnO_2_‐motors. The motion of nanomotors was analyzed with ImageJ for 10 s (*n* = 52). i) Ultrasound detection of the generated oxygen in vitro.

After confirming the O_2_ generation property of MnO_2_‐motors, the autonomous movement of our nanomotors was recorded by a Nikon Ti2‐A inverted optical microscope under PBS and simulated synovial fluid (SSF). Image J with manual tracking module was then used to further analyze the trajectory path (**Figure** **3**a–e; Figure S5 a–c and Movie S1, Supporting Information) and the motion characteristics including average velocity and accumulated distance. As demonstrated in Figure 3a–e, enhanced diffusion of our MnO_2_‐motors was observed in the presence of H_2_O_2_ solutions with a concentration‐dependent manner. Obviously, powered by H_2_O_2_ fuel, the speed of MnO_2_‐motors increased gradually along with H_2_O_2_ concentration whereas a typical Brownian motion was recorded under H_2_O_2_‐free condition. The average speed of nanomotors reached 4.3 µm s^−1^ under 10 mm H_2_O_2_ solution, which was 1.7 times higher than that without H_2_O_2_ addition (Figure 3f). Normally, the concentration of H_2_O_2_ under arthritic microenvironment varies, approximately ranging from 0.1–1 m, depending on the severity of inflammation.^[^ ^26^ ^]^ Surprisingly, our MnO_2_‐motors also presented similar motion pattern under SSF with a speed up to 3.4 µm s^−1^ at the presence of 1 m H_2_O_2_ (Figure S5, Supporting Information).

The mean square displacements (MSDs) of MnO_2_‐motors were also analyzed according to the self‐diffusiophoretic model proposed by Golestanian et al.^[^ ^27^ ^]^ The fitting curve depicted in Figure 3h; Figure S5f, Supporting Information, shows that the MSDs of MnO_2_‐motors increased with the higher fuel concentration and such motion behavior would greatly improve the diffusion efficiency of the produced O_2_ in H_2_O_2_‐rich microenvironment and therefore relieve the hypoxic conditions in an on‐demand manner.

To further investigate the echogenic properties of O_2_‐releasing MnO_2_‐motors, water‐sac phantom tests were then carried out. H_2_O_2_ solutions with different concentrations were used to simulate the inflammation microenvironment in arthritis. The concentration of H_2_O_2_ under arthritic microenvironment varied, approximately ranging from 0.1 to 1 mm, depending on the severity of inflammation.^[^ ^26^ ^]^ According to Figure 3i; Figure S6, Supporting Information, the echogenic signal of O_2_ was clearly detected even under 0.1 mm H_2_O_2_ solution. With the H_2_O_2_ concentration raised to ten times higher, which simulated the highest H_2_O_2_ concentration of in vivo inflammation condition as ever reported,^[^ ^28^ ^]^ more O_2_ were generated, displaying higher echogenic signal (Movie S2, Supporting Information). Collectively, O_2_ generation from MnO_2_‐motors is detectable under ultrasound imaging and may be promising for inflammatory disease identification.

### 2.3 In Vitro ROS Scavenging and Inflammation Attenuation

**Figure 4:**
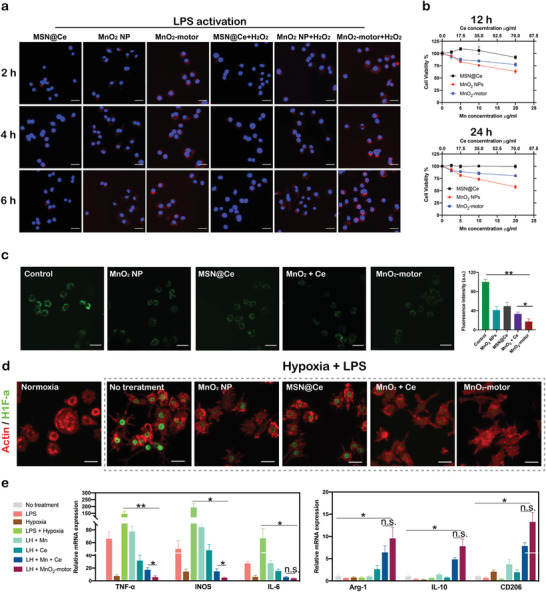
In vitro evaluation of MnO_2_‐motors. a) Intracellular uptake of MnO_2_‐motors with or without H_2_O_2_ for 2, 4, and 6 h. Scale bar = 50 µm. b) Cell viability as determined by CCK‐8 assays after incubation with MnO_2_ NPs, MSN@Ce, and MnO_2_‐motors at various concentrations for 12 or 24 h (*n* = 4; mean ± SD). c) Inverted fluorescence microscopy images and corresponding fluorescence intensity of intracellular H_2_O_2_ in RAW264.7 cells incubated with MnO_2_ NPs, MSN@Ce, MnO_2_ + Ce, and MnO_2_‐motors in the presence of H_2_O_2._ Data represent mean ± SD (*n* = 4). Scale bar = 50 µm. d) HIF‐1*α* staining of RAW 264.7 cells pretreated with MnO_2_ NPs, MSN@Ce, MnO_2_ + Ce, and MnO_2_‐motors for 2 h and subsequently incubated for 8 h under hypoxic and inflammatory conditions. Scale bar = 50 µm. e) mRNA expression of M1 and M2 macrophage markers in RAW264.7 cells under various conditions, as evaluated by qRT‐PCR analysis. Data represent mean ± SD (*n* = 3). In (c,e), data analyzed using one way ANOVA test. (*p < 0.05, ** *p* < 0.01)

Macrophages in the RA synovial microenvironment are considered to play pivotal roles in inflammation progression,^[^ ^2a^ ^]^ which is an effective target of ROS‐scavenging treatment. Upon stimulation with LPS, RAW264.7 cells undergo M1 polarization through ROS production, including H_2_O_2_, ^•^OH, and O^2•−^.^[^ ^29^ ^]^ To further illustrate the enhanced diffusion of our motors, 0.1 mm H_2_O_2_ was then adopted as the extra fuel in the cell uptake experiment. As shown in **Figure** **4**a; Figure S7, Supporting Information, under LPS activation, MnO_2_‐motors treated RAW264.7 cells exhibited much stronger fluorescence compared to the cells incubated with MSN@Ce as well as MnO_2_ NPs. In addition, MnO_2_‐motors were taken up quickly by LPS‐treated RAW264.7 macrophages within 2 h and the cellular accumulation of nanomotors was not affected by the extra addition of H_2_O_2_ fuel. The results obtained herein demonstrated that MnO_2_‐motors possessed excellent cellular uptake capability due to the enhanced diffusion under H_2_O_2_ substrate, and thereby permeated cellular membranes to scavenge ROS and relieve hypoxia effectively. Furthermore, as determined by CCK‐8 assay (Figure 4b), MnO_2_‐motors and MSN@Ce did not exhibit cytotoxicity after 12 or 24 h incubation. The viability of RAW264.7 cells was slightly affected by co‐culturing with MnO_2_ NPs for 24 h at high Mn concentration (20 µg mL^−1^), indicating that ceria NPs may scavenge the toxic hydroxyl radicals produced by MnO_2_ NPs. Thus, based on these results and previous reports^[^ ^26^ ^]^ regarding Mn‐based NPs for disease treatment, a concentration of MnO_2_‐motors containing 10 µg mL^−1^ of Mn was used in the following experiments.

To verify the ROS scavenging effect of MnO_2_‐motors, intracellular H_2_O_2_‐degradtion was first investigated with an intracellular H_2_O_2_ assay, a fluorescent probe specific for H_2_O_2_ detection. The intracellular H_2_O_2_ levels were evaluated by inverted fluorescence microscope after incubating with 100 µm H_2_O_2_ for 1 h. Compared with MnO_2_ NPs and MSN@Ce group, MnO_2_‐motors exhibited much lower fluorescence intensity and decomposed intracellular H_2_O_2_ more efficiently, demonstrating the synergistic effect of ceria and MnO_2_ NPs. (Figure 4c). In addition, the fluorescence intensity of MnO_2_ + Ce group (containing the same Mn and Ce concentration) was also significantly higher than that of our MnO_2_‐motors, confirming the outstanding H_2_O_2_‐degrading effect of our nanomotors due to substrate‐actuated enhanced diffusion. We then evaluated the ROS levels of LPS‐stimulated RAW 264.7 cells using 2′,7′‐dichloroflfluorescin diacetate (DCFH‐DA), a ROS indicator sensitive to hydroxyl radicals. After activation by LPS, RAW264.7 cells showed deepened fluorescence significantly, indicating the increase of ROS production levels, which have high correlation with the severity of inflammation in diseased sites. According to Figure S8, Supporting Information, MnO_2_‐motors exhibited superior therapeutic effect by reducing the LPS‐stimulated overproduction of hydroxyl radicals dramatically whereas relatively weak inflammation scavenging capability was observed for the non‐motor group (MnO_2_ + Ce). The autonomous motion endows the MnO_2_‐motors higher diffusion intracellularly and thus results in a better active ROS scavenging efficacy. The hypoxic microenvironment in RA joints normally induces HIF‐1*α* expression, which is also reported to affect the balance of macrophage subtypes.^[^ ^7b^ ^]^ To verify the hypoxia‐attenuating ability of MnO_2_‐motors, HIF‐1*α* expression levels were then evaluated by immunofluorescence staining. After pretreatment with MnO_2_ NPs, MSN@Ce, MnO_2_ + Ce, and MnO_2_‐motors, LPS‐activated RAW264.7 cells were incubated under a hypoxic chamber for 8 h. According to Figure 4d, hypoxia and LPS induced HIF‐1*α* expression in RAW264.7 cells successfully, which was attenuated by the treatment with MnO_2_ NPs, MSN@Ce, MnO_2_ + Ce, and MnO_2_‐motors. As we expected, MnO_2_‐motors exhibited the most prominent HIF‐1*α* inhibition efficacy, which reflected the active delivery capability of MnO_2_‐motors, leading to enhanced O_2_ diffusion. The O_2_ indicator was then used to detect the O_2_ generation efficiency at intracellular level. As shown in Figure S9, Supporting Information, MnO_2_ NPs, MSN@Ce, and MnO_2_ + Ce could relieve the hypoxic condition to some extent, while the intracellular O_2_ level was significantly upregulated in MnO_2_‐motors‐treated cells, further revealing the enhanced O_2_ generation effect of MnO_2_‐motors.

Macrophages in RA joints are primarily M1 phenotype, promoting RA progression by releasing various inflammatory cytokines. Real Time‐PCR and Western blot tests were further carried out to study the anti‐inflammatory effect of MnO_2_‐motors by suppressing the activation of M1 type macrophages. Upon stimulation with LPS and hypoxia, RAW264.7 cells underwent M1 polarization, secreting pro‐inflammatory cytokines such as iNOS, TNF‐*α*, and IL‐6. As shown in Figure 4e, MnO_2_‐motors markedly down‐regulated the expression levels of M1 markers, including iNOS, TNF‐*α*, and IL‐6, suggesting that our MnO_2_‐motors possessed remarkable capacity in blocking M1 polarization through mitigating inflammation responses. In contrast, the anti‐inflammatory M2 markers including Arg‐1, CD206, and IL‐10 were greatly increased after MnO_2_‐motors treatment, mediating effective M1 to M2 polarization effect. In Western blot analysis, LPS + hypoxia condition mediated M2‐M1 polarization with remarkably enhanced expression of TNF‐*α* and IL‐1*β*, decreased expression of Arg‐1 and IL‐10, and further induced the expression of HIF‐1*α* (Figure S10, Supporting Information). As shown in Figure S10, Supporting Information, MnO_2_ + Ce treatment could indeed inhibit M1 polarization and relieve hypoxia as indicated by the suppressed expression of TNF‐*α*, IL‐1*β*, and HIF‐1*α*. However, MnO_2_‐motors treatment remarkably reduced all these negative effects, even correcting them to approximately normal levels. These findings suggested the credible HIF‐1*α* inhibition and macrophages remodulation of MnO_2_‐motors under arthritic microenvironment, highlighting the superiority of enhanced diffusion via MnO_2_‐motors, and thereby possibly presenting higher anti‐inflammation effect in RA treatment.

### 2.4 In Vivo Active Arthritic Regulation Therapy Performances of MnO_2_‐Motors

**Figure 5:**
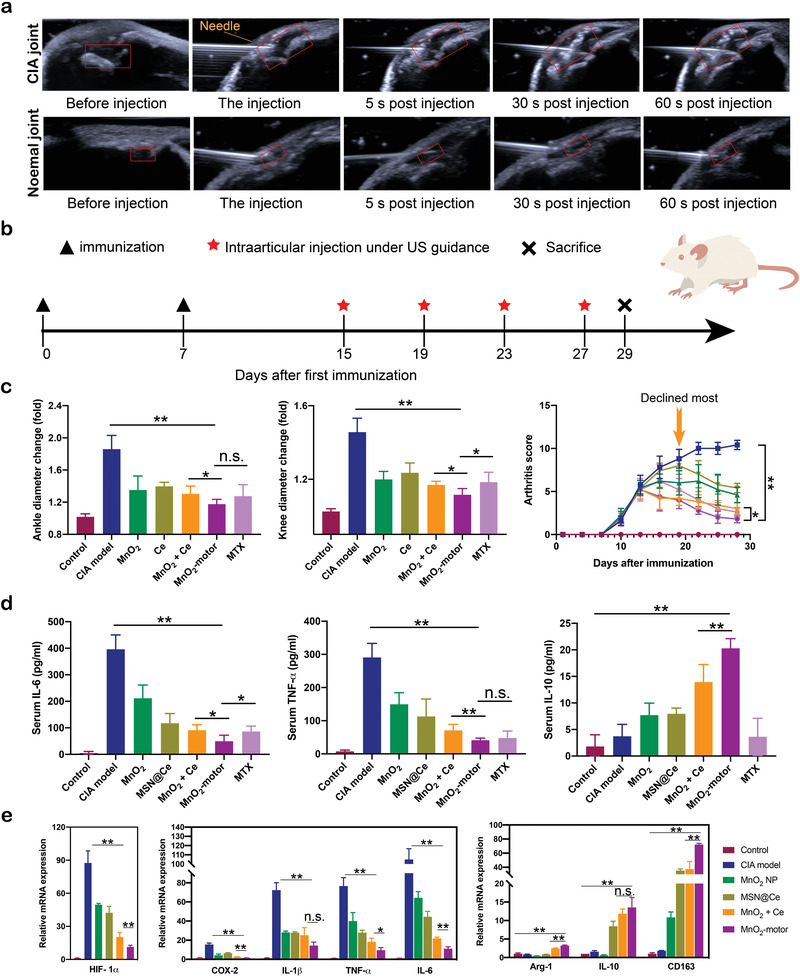
In vivo therapeutic effects of MnO_2_‐motors. a) Ultrasound images of the in vivo detection in a CIA joint as compared with a normal joint (the articular cavity is marked by a red box). b) The study protocol of a therapeutic regimen with a CIA rat model. c) Joint diameter and clinical score of CIA model rats after different treatments. Data represent mean ± SD (*n* = 5). d) Quantitative analysis of TNF‐*α*, IL‐10, and IL‐6. Data represent mean ± SD (*n* = 5). e) qRT‐PCR analysis of HIF‐1*α* and M1 and M2 macrophage markers in synovial tissue. Data represent mean ± SD (*n* = 3). In (c,d,e), data analyzed using one‐way ANOVA test. (*p < 0.05, **p < 0.01)

To investigate the therapeutic effectiveness of MnO_2_‐motors for RA treatment, CIA rat model based on the injection of the mixture of type 2 collagen and complete Freund's Adjuvant was established. It was reported that the important pathologic features of human RA, including chronic synovitis and bone destruction, are shared in the CIA model.^[^ ^30^ ^]^ Using the rat CIA model, we first visualized the oxygen generation through ultrasound imaging. Ultrasound imaging is widely used for precise diagnosis of synovitis, chondral lesions, and subchondral bone damage.^[^ ^31^ ^]^ As shown in **Figure** **5**a, the thickened synovium, joint effusion, and the changes of periarticular soft tissues in CIA rats were clearly observed by ultrasound. In healthy rats, the thickness of articular cartilage was normal and without arthredema. After injecting MnO_2_‐motors, an echogenic focus of the articular cavity area (red box) was observed immediately in CIA rat (Figure 5a; Movie S3, Supporting Information), which meant that large amount of O_2_ was produced in the H_2_O_2_‐rich joint fluid, revealing the arthritis severity of the CIA rat. Furthermore, obvious ultrasonic signal enhancement lasted for at least 60 s after the injection, indicating the availability for disease assessment. The administration of MnO_2_‐motors into a healthy rat was used as a control and no echogenic signal enhancement was observed. Based on our results, the utilization of MnO_2_‐motors could generate oxygen locally and enhance echogenic signal, which is helpful for joint progression assessment and accurate diagnosis.

As the MnO_2_‐motors were injected intra‐articularly, the biodistribution of the MnO_2_‐motors in joints was further verified in vivo. The CIA rats received an injection of free Rhodamin B solution, Rhodamin B ‐labeled MnO_2_ NP, and Rhodamin B ‐labeled MnO_2_‐motor and were then subjected to in vivo imaging. As shown in Figure S11, Supporting Information, almost no fluorescence signals were observed in the joints of the free Rhodamin B after 24 h post‐administration. The MnO_2_ NP could sustain in the joint to some extent, which might be attributed to the passive reaction between MnO_2_ and joint fluid. In contrast, due to the enhanced diffusion, the MnO_2_‐motor presented the most persistent fluorescence intensity in the joint, retaining for more than 24 h, which was obviously longer than that of MnO_2_ NP.

The effectiveness of MnO_2_‐motors with various control groups including untreated CIA rat, MnO_2_ NPs, MSN@Ce, MnO_2_ + Ce, and methotrexate (MTX) was also compared. MTX is widely used for alleviating RA;^[^ ^32^ ^]^ and therefore, was considered as a positive control. MnO_2_ NPs, MSN@Ce, MnO_2_ + Ce, MnO_2_‐motors, and MTX were injected intra‐articularly twice a week from day 15 until the study endpoint (day 29). (Figure 5b) The arthritis severity in clinical evaluation was determined by clinical arthritis score, paw thickness, and joint diameter. Compared with other groups, the arthritis score of MnO_2_‐motors treated group declined most after 5 days of treatment and the swollen paws shrunk considerably (Figure 5c), implying that the inflammation was effectively controlled. Frankly, RA patients were often accompanied with weight loss as RA is a chronic wasting disease, which is consistent with our CIA rat model. A moderate increase was observed in the rest of the therapy groups after day 15 post‐induction (Figure S12, Supporting Information).

Furthermore, to verify the effect of MnO_2_‐motors on the production of inflammatory cytokines which are closely associated with RA development, the serum levels of TNF‐*α*, IL‐6, and IL‐10 were then determined. As shown in Figure 5d, the MnO_2_ + Ce and MnO_2_‐motors treatment suppressed the pro‐inflammatory cytokines (TNF‐*α* and IL‐6) while up‐regulating the anti‐inflammatory cytokines (IL‐10) in macrophages but neither individual MnO_2_ NP treatment nor individual MSN@Ce injection did, indicating that the therapeutic effect of MnO_2_‐motors in RA may result from the regulation of macrophage phenotypic transition by simultaneously enhancing the anti‐inflammatory macrophages and reducing the pro‐inflammatory macrophages. Similarly, M1 macrophage specific biomarkers in joint synovium such as Cox‐2, TNF‐*α*, IL‐1*β*, and IL‐6 were increased after inducing CIA, whereas MnO_2_‐motors injection remarkably reduced their levels. Compared with MnO_2_ + Ce group, MnO_2_‐motors accompanied with enhanced diffusion additionally up‐regulated the expression levels of anti‐inflammatory M2 markers, Arg‐1, IL‐10, and CD163 to a more remarkable level. Noticeably, acting as O_2_ generators, our MnO_2_‐motors also alleviated hypoxia in CIA knee joints with markedly attenuating the HIF‐1*α* expression in joint synovium (Figure 5e).

**Figure 6:**
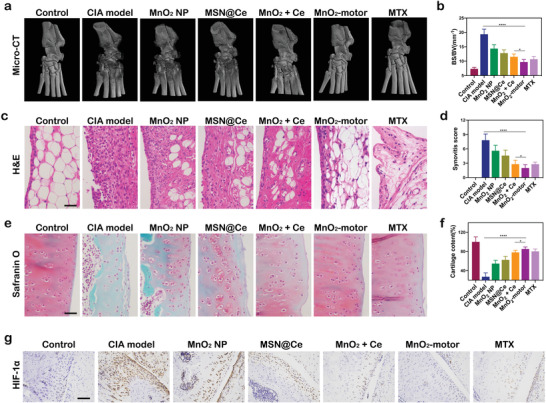
In vivo assessments of pathological features of CIA rat models. a) Representative Micro‐CT images of joint at day 29 after different treatments. b) Ratio between bone surface and bone volume. Data represent mean ± SD (*n* = 3). c) Hematoxylin and eosin (H&E) staining of synovium. Scale bar = 100 µm. d) Synovitis score. Data represent mean ± SD (*n* = 5). e) Safranin‐O staining of cartilage of CIA joints. Scale bar = 100 µm. f) Cartilage content. Data represent mean ± SD (*n* = 5). g) Immunohistochemical evaluation of HIF‐1*α* on knee sections. (*n* = 5). Scale bar = 200 µm. In (b,d,e), data analyzed using one‐way ANOVA test. (*p < 0.05, **p < 0.01)

At the peak of arthritis, the rats showed tri‐legged gait, and severe edema in the tissue. From Figure S14, Supporting Information, it is seen that neither individual MnO_2_ NP injection nor MSN@Ce obviously influenced the deterioration of arthritis in the treatment process. Surprisingly, erythema and paw swelling were dramatically relieved in rats treated with MnO_2_ + Ce or MTX and further reduced after MnO_2_‐motors treatment, suggesting the credible curative effect of MnO_2_‐motor due to the enhanced diffusion under inflamed‐arthritic microenvironment (Figure S14, Supporting Information). As bone damage, cartilage destruction, and synovial infiltration are the main features of RA progression, micro‐CT, histological, and immunohistochemical analyses were employed to verify the effect of MnO_2_‐motors. In **Figure** **6**a, the CIA model group displays rough bone surfaces in the joints, whereas the MnO_2_‐motors treated group presents a better change in bone erosion with markedly reduced ratio between bone surface and bone volume (Figure 6b), suggesting a successful inhibitory effect of bone damage. Furthermore, synovial inflammation was significantly attenuated in MnO_2_‐motors and MTX injection groups as evaluated by hematoxylin and eosin (H&E) staining (Figure 6c,d). In addition, Safranin‐O staining of the knee joint showed complete destruction of cartilage in model group. In contrast, the injection of MnO_2_‐motors group exhibited much clearer boundaries of cartilage than MnO_2_ + Ce group, comparable to those of the healthy group. (Figure 6e,f) This suggested the potent effect of MnO_2_‐motor for RA treatment due to the enhanced diffusion by consuming the local hydrogen peroxide in the joints. Finally, the up‐regulated expression of HIF‐1*α* is a typical response triggered by hypoxia for increasing oxygen demand. Surprisingly, the number of HIF‐1*α* immuno‐active cells was remarkably reduced in MnO_2_‐motors, MnO_2_ + Ce, and MTX treatment groups. Among them, MnO_2_‐motors showed more efficient oxygen supplement (Figure 6g), owing to enhanced local catalytic H_2_O_2_ consumption by resolving the oxygen demand of fibroblast‐like synoviocyte hyperplasia and infiltrated inflammatory cells. After the treatment, hemolysis assay was further carried out to evaluate the safety. According to Figure S15, Supporting Information, our MnO_2_‐motors had a negligible hemolysis at different concentrations, demonstrating that the application of MnO_2_‐motors was feasible and ready for clinical practice.

## 3 Conclusion

Here, we report the potential for an arthritic microenvironment powered nanomotor system that simultaneously integrates hypoxia relief and ROS scavenging for macrophages phenotypic regulation. This study demonstrates the efficacy and feasibility of an ultrasound‐guided in situ inflammation detector in a CIA rat model, where both pathophysiology and administration mimic what would be observed and performed in humans. This is the pioneered example to our knowledge of adopting the H_2_O_2_‐fueled O_2_ releasing behavior for RA joint progression assessment together with on‐demand therapy. In comparison with the preexisting nanomedicine for RA therapy, our nanomotors with enhanced diffusion displayed on‐demand releasing behavior due to the typical Janus structure and catalytic activity for H_2_O_2_ decomposition, facilitating the application scope to be extended to clinical practice in successive work.

In summary, MnO_2_‐motors were successfully developed via pickering emulsion that could be easily modified with high yields for RA detection and therapy. By decomposing the overproduced H_2_O_2_ under arthritic microenvironment, active MnO_2_‐motors decorated with ceria NPs showed synergistic effect on efficient oxygen generation and also alleviated inflammation through macrophages modulation. Thanks to the local H_2_O_2_ actuated enhanced diffusion, our MnO_2_‐motors further exhibited continuous oxygen releasing behavior both in vitro and in vivo, successfully postponing the progression of CIA joints by inhibiting the pro‐inflammatory phenotype of macrophages as well as relieving the hypoxic condition. As a powerful and active O_2_ generator and ROS scavenger, our MnO_2_‐motor is promising for detection and treatment of hypoxia‐related inflammation diseases.

## 4 Experimental Section

### Materials

Chemical reagents were purchased as follows: Cerium (III) acetate sesquihydrate, (C_6_H_12_CeO_7.5_, 99%), oleylamine (70%), xylene (99.5%), tetraethyl orthosilicate (TEOS), and chloroform were purchased from Sigma–Aldrich. mPEG‐DSPE was purchased from Yarebio. Hoechst 33342 and 2′,7′‐dichloroflfluorescin diacetate (DCFH‐DA, 97%) were bought from Beyotime Biotechnology. IL10 Rabbit Polyclonal Antibody, Arginase‐1 Mouse Monoclonal Antibody, TNF alpha Mouse Monoclonal Antibody, IL1 beta Rabbit Polyclonal Antibody, and Alexa Fluor 488‐labeled secondary antibody were purchased from Proteintech Group (Wuhan, China). H1F‐1*α* Rabbit Polyclonal Antibody and IL‐10 Rabbit Polyclonal Antibody were purchased from Abcam. SYBR Green Realtime PCR Master Mix and the ReverTra Ace qPCR RT Kit were obtained from Toyobo Co. Japan. All reagents for cell culture were bought from Gibco. The purified deionized water was prepared by the Milli‐Q plus system (Millipore, USA). All other chemical regents used in this experiment were analytically pure without further purification.

### Synthesis of Ceria NPs

Ceria NPs were synthesized using a reported method.^[^ ^16^ ^]^ First, Cerium (III) acetate (1 mmol) and 12 mmol of oleylamine were dissolved in 15 mL of xylene with continuous stirring for 2 h at room temperature. Then, the resulting solution was aged at 90 °C for 3 h and cooled to room temperature after 1 mL of deionized water was added. The resulting ceria NPs were washed with ethanol several times and finally redispersed in chloroform.

Subsequently, for in vivo application with better biocompatibility, 5 mL of ceria NPs solution in chloroform was added into 5 mL of mPEG‐DSPE solution (20 mg mL^−1^ in chloroform) and then stirred for 10 min. The following mixture was incubated at 60 °C under vacuum in a rotary evaporator for 1 h. After being cooled down, 5 mL of deionized water was added and then sonicated for 15 min to obtain PEGylated ceria NPs. After that, the mixture was collected by ultra‐centrifugation at 20 000 rpm for 30 min and finally dispersed in deionized water.

### Synthesis of Large Pore‐Sized MSNs NPs and MSN@Ce

MSNs were synthesized according to the previous report^[^ ^23^ ^]^ with several modifications. Briefly, 0.5 mL chloroform containing iron oxide nanoparticles (6.0 mg Fe per mL as determined by ICP‐MS) was mixed into 10 mL of 0.055 m CTAB aqueous solution and the mixture was vigorously stirred for 30 min to form oil‐in‐water microemulsions. Then, to evaporate the chloroform, the mixture was heated to 60 °C and stirred for 10 min. The resulting solution was added into a mixture of 95 mL deionized water (DW), 5 mL methanol, 20 mL ethyl acetate, and 3 mL ammonium hydroxide solution with continuous stirring. Then, 2 mL of TEOS was added dropwise to the reaction solution and stirred for 12 h at room temperature to initiate the silica sol–gel reaction. The as‐synthesized MSNs were washed three times with excess ethanol. For the extraction of the CTAB, the pH of the as‐synthesized MSNs in ethanol was adjusted to pH 1.6 by adding diluted HCl solution and the solution was stirred for 3 h at 60 °C. To synthesize amine‐functionalized MSNs, 0.15 mL of APTES was added to the MSN solution and heated to 70 °C for 3 h. The final product was obtained by washing several times with ethanol.

MSN@Ce was then prepared by adding 60 mg of ceria NPs into 20 mg of amine‐functionalized MSNs with overnight stirring at room temperature. Finally, the product was washed with ethanol and water several times and redispersed in 5 mL of deionized water.

### Fabrication of MnO_2_‐Motor

MnO_2_ motors were prepared with pickering emulsions method. Briefly, 0.5 g of wax was added into 5 mL of MSN@Ce solution in deionized water and the mixture was heated at 80 °C to ensure a low viscosity of the liquid wax. Emulsions were obtained using high speed homogenizer stirring at 15 000 rpm for 2 min and then cooled down to room temperature. The obtained MSN@Ce/solid wax droplets were filtered using low speed filter paper and rinsed with deionized water to remove free or weakly attached MSN@Ce particles from solid wax droplets.

After that, 1 g of solid wax droplets embedded with MSN@Ce was added to 10 mL potassium permanganate solution (KMnO_4_) (20 mg mL^−1^) and then stirred overnight. Slow speed filter paper was then used to remove the unreacted KMnO_4_. The precipitation was taken and centrifuged with ethanol and chloromethane for three times, respectively. The resulting MnO_2_ nanomotors were obtained after cleaning centrifugation with ethanol followed by deionized water for several times.

The passive control MnO_2_ NPs were simply fabricated by adding the amine‐functionalized MSN into KMnO_4_ solution and stirring overnight, followed by cleaning centrifugation procedure.

### Characterization of MnO_2_‐Motor

The morphology of MnO_2_‐motors was analyzed by a JEM 1400 transmission electron microscope with an acceleration voltage of 120 kV (JEOL, Japan). Scanning electron microscopy (SEM) and energy‐dispersive X‐ray spectroscopy (EDS) were performed using a Phenom scanning electron microscope. Metal ion concentration including manganese and ceria was analyzed by inductively coupled plasma atomic emission spectroscopy (ICP‐MS, NexION 2000B, PerkinElmer). Hydrodynamic diameter, polydispersity, and zeta potential of motors were measured using a dynamic light scattering instrument (ZSE, Malvern).

### H_2_O_2_ Decomposition and O_2_ Generation Assay

MnO_2_ NPs, MSN@Ce, and MnO_2_‐motor containing different amounts of manganese and ceria were added into 2.5 mm H_2_O_2_ at room temperature. Then, the H_2_O_2_ concentration was measured with a H_2_O_2_ assay kit (Bestbio). MnO_2_ NPs, MSN@Ce, and MnO_2_‐motors containing different amounts of manganese and ceria was mixed in 10 mm H_2_O_2_ at room temperature, followed by measuring the O_2_ concentration using a dissolved oxygen meter oxygen probe (JPBJ‐608 portable, Shanghai REX Instrument Factory).

### ROS Scavenging Activity Assay

Two main ROS, O^2•−^, and •OH, were used to evaluate the ROS scavenging capability of MnO_2_‐motors. The superoxide anion scavenging activity was conducted with a SOD assay kit (Sigma–Aldrich, USA). Scavenging activity of hydroxyl radical was measured with a hydroxyl radical antioxidant capacity (HORAC) assay kit (Cell Biolabs, Inc., USA).

### Movement Recording and Analysis

A Nikon Ti2‐A inverted optical microscope equipped with a high‐speed camera (sCMOS) and NIS Elements AR3.2 software was used to record the motion behavior of nanomotors. The motion trajectories of the nanomotors under different H_2_O_2_ concentrations (0, 0.1, 1, 5, and 10 mm for PBS and 0, 0.1, and 1 mm for SSF) were recorded. SSF were obtained by adding hyaluronic acid (0.3%) into PBS as reported.^[^ ^33^ ^]^ The motion was recorded for 10 s and at least 52 nanomotors were analyzed under each condition. Subsequently, the tracking image sequences and the speed of the nanomotors were analyzed by ImageJ plugin manual tracking according to the previous reports. Mean‐square‐displacement (MSD) analysis was conducted by using the self‐diffusiophoretic model proposed by Golestanian et al.^[^ ^27^ ^]^

### Ultrasonographic Imaging of MnO_2_‐Motors

In vitro ultrasound imaging was performed under B mode (ACUSON Sequoia, SIEMENS, Germany), with an 18 MHz transducer. H_2_O_2_ solutions with different concentrations (0, 0.1, 1, and 10 mm) were used for the measurement. A hand‐made water sac phantom was used to simulate the arthritis inflammation environment and MnO_2_‐motors suspension was injected via a three‐port valve. Then, the change of echogenic signal was recorded. The mean gray value of ultrasound images was then measured by Image J.

### Cell Culture and Cell Uptake

RAW264.7 cells were cultured in DMEM with 10% heat inactivated FBS and antibiotics at 37 °C in a humidified atmosphere with 5% CO_2_. RAW264.7 cells were seeded onto the 96‐well plates with a density of 8 × 10^3^ cells per well and incubated for 24 h. After culturing with LPS (1 µg mL^−1^) for 6 h, the medium was replaced by rhodamine B‐labeled MnO_2_‐motors, MSN@Ce, and MnO_2_ NP with equal Mn and Ce concentration (10 µg Mn per mL, 35 µg Ce per mL). The cellular uptake of MnO_2_‐motors in the presence or absence of H_2_O_2_ was investigated, respectively. After incubation for 2, 4 and 6 h, the cells were washed and stained with Hoechest for 15 min. Then, the intracellular uptake of nanomotors was captured by fluorescence microscopy.

### In Vitro Cell Viability and Intracellular ROS Level

The cytotoxicity of MnO_2_ NPs, MSN@Ce, and MnO_2_‐motors (containing equal amounts of Mn) was tested with RAW264.7 cells. Cells were seeded at 1 × 10^4^ cells per well in 96‐well plates. 24 h later, the cells were incubated with MnO_2_ NPs, MSN@Ce, and MnO_2_‐motors. After incubation for another 12 or 24 h, the cell viability was quantified using a CCK‐8 assay kit (Beyotime Biotechnology), respectively.

### Intracellular H_2_O_2_ Assay

An intracellular H_2_O_2_ assay (Bestbio) was used to assess the intracellular H_2_O_2_ concentration. 100 µm of H_2_O_2_ was used to treat RAW264.7 cells, which were pre‐incubated with 40 µg mL^−1^ of MnO_2_ NPs, MSN@Ce, MnO_2_ + Ce, and MnO_2_‐motors for 24 h. After 1 h, the cell media were replaced with assay buffer, and the intracellular H_2_O_2_ concentration was examined by fluorescence microscopy.

### Intracellular ROS Evaluation

Intracellular ROS levels of RAW264.7 cells were analyzed with DCFH‐DA (Beyotime, China). Cells were incubated with lipopolysaccharide (LPS, 1 µg mL^−1^) and samples (MnO_2_ NPs, MSN@Ce, MnO_2_ + Ce, and MnO_2_‐motors containing equal amount of Mn and Ce) for 12 h. Thereafter, 20 µm DCFH‐DA was then added and incubated for another 30 min. The fluorescence intensity was instantaneously analyzed by Multifunctional Microplate Reader (TECAN, San Jose, CA), with an excitation wavelength of 488 nm and an emission wavelength of 525 nm. In addition, the green fluorescence intensity of the well was also examined under the fluorescence microscopy.

### HIF‐1*α* Immunostaining

HIF‐1*α* immunostaining was carried out under hypoxic condition in an LPS‐containing media. Hypoxic condition was achieved by incubation with AnaeroGen (Thermo Scientific) for 8 h. RAW264.7 cells were pre‐incubated with 40 µg mL^−1^ of MnO_2_ NPs, MSN@Ce, MnO_2_ + Ce, and MnO_2_‐motors for 2 h, followed by adding 1 µg mL^−1^ of LPS under hypoxic conditions for another 8 h. Thereafter, the cells were stained with primary antibody against HIF‐1*α* (Abcam) and Alexa Fluor 488‐labeled secondary antibody (Proteintech). F‐actin was co‐stained using rhodamine phalloidin (Soalrbio).

### Intracellular O_2_ Evaluation

The O_2_ generation efficiency in cells was investigated with an O_2_ sensing probe, [(Ru(dpp)_3_)]Cl_2_ (Sigma–Aldrich, Co. Ltd.). The fluorescence of this agent could be strongly quenched by O_2_. RAW264.7 cells were cultured at a density of 1 × 10^4^ cells per well in 96‐well plate and under hypoxia condition for 24 h. Then, the cells were incubated with O_2_ sensing probe for 4 h and with 40 µg mL^−1^ of MnO_2_ NPs, MSN@Ce, MnO_2_ + Ce, and MnO_2_‐motors for another 4 h. The fluorescent signal of [Ru(dpp)_3_]Cl_2_ in cells was then observed and photographed under inverted fluorescence microscopy.

### qRT‐PCR and Western Blot Analysis

RAW264.7 cells were pretreated with MnO_2_ NPs, MSN@Ce, MnO_2_ + Ce, and MnO_2_‐motors for 2 h, followed by extensive PBS washing. Fresh DMEM supplemented with LPS (1 µg mL^−1^) was added and the cells were incubated in hypoxic condition. After 8 h, total RNA and proteins from RAW264.7 cells were extracted with Super Total RNA Isolation Kit (Foregene) and cell lysis buffer (Cell Signaling Technology), respectively, according to the manufacturer's instructions. RNA Isolation Kit was used to isolate the total RNA, which was reverse transcribed into cDNA according to the standard protocols with a cDNA ReverTra Ace qPCR RT Kit (Toyobo). Real‐time PCR was carried out with SYBR Green Realtime PCR Master Mix (Toyobo) on a LightCycler 480 Instrument (Roche). PCR steps are described as follows: Initial denaturation at 95 °C for 10 min, denaturation at 95 °C for 15 s, and annealing/extension at 60 °C for 1 min for 40 cycles. The relative expression of mRNA was quantified using 2^−ΔΔ^Ct method.

To perform Western blot analysis, BCA assay was carried out for measurement of protein concentration. Proteins were then mixed with NuPAGE lithium dodecyl sulfate (LDS) sample buffer (Life Technologies), boiled at 95 °C for 10 min for denaturation, and loaded on 10% (w/v) sodium dodecyl sulfate polyacrylamide gel. After 40 min of electrophoresis at 150 V, proteins were transferred to nitrocellulose membrane (Bio‐Rad) using a Trans‐Blot SD wet electrophoretic transfer cell (Bio Rad) for 70 min at 120 V. To avoid non‐specific binding, membranes were then blocked with 5% BSA for 1 h. Next, membranes were probed with primary antibodies; HIF‐1*α* and IL‐10 (Abcam), Arg‐1, IL‐1*β*, and TNF‐a (Proteintech) overnight at 4 °C. The next day, membranes were washed with TBS‐T three times and reacted with HRP‐conjugated secondary antibodies for 60 min. After washing, membranes were then developed using chemiluminescence detection system (FluorChem R).

### Animal Model Induction and Treatment

Male Sprague‐Dawley rats (270 ± 20 g) were brought from Changsheng Biotechnology. All the animal procedures were carried out under the guideline approved by the Institutional Animal Care and Use Committee (IACUC) of the Southern Medical University (permit number: SYXK 2016‐0041). CIA model was established by injecting 200 µL of emulsified solution (1:1) mixing type 2 collagen (2 mg mL^−1^) and complete Freund's Adjuvant (CFA) (1 mg mL^−1^) into male SD rat and injecting another 100 µL of emulsified solution (1:1) mixing type 2 collagen (2 mg mL^−1^) and CFA (1 mg mL^−1^) into the tail 7 days later intradermally. The rats were randomly assigned after first signs of inflammation observed at day 10. The treatment began at day 15 with seven groups (*n* = 5). The healthy rats were applied as the normal control while the CIA rats without treatment were the model rats. 100 µL of PBS, MnO_2_ NPs, MSN@Ce, MnO_2_ + Ce, MnO_2_‐motors, and MTX were administered via intraarticular injection at the knee joint. MnO_2_ NPs, MSN@Ce, MnO_2_ + Ce, and MnO_2_‐motors were injected at a 10 µg dose of manganese and 35 µg of ceria, as determined by ICP‐MS analysis. Positive control rats were injected with MTX (5 µg in 100 µL PBS) on the same days. All intra‐articular injections were performed under ultrasound guidance in real time. Images were then assessed for changes in signal at the injection site with an 18 MHz transducer under B‐mode ultrasound. (ACUSON Sequoia, SIEMENS, Germany)

### Joint Biodistribution

For in joint biodistribution, Rhodamin B (RhB) was added into the solution of MnO_2_‐motors and stirred overnight to obtain RhB@ MnO_2_‐motors. Then, CIA model rats were injected intra‐articularly with 200 µL free RhB and RhB@ MnO_2_‐motors in PBS, respectively. All rats were gaseous anesthetized and the in vivo fluorescence imaging was recorded by small animal imaging system (excitation/emission = 920/980 nm) at 1, 6, 12, and 24 h post‐administration.

### Joint Swelling Measurements and Clinical Scores

The diet, mental state, fur color, walking gait, and joints swelling of the rats were observed every day. The weight and joint diameter of each rat were measured once every 3 days during the disease progression from day 1. Ankle and knee joint from medial to lateral were detected using a digital caliper. Thickness of each hind paw was also measured to further evaluate the joint swelling degree. Arthritis scores of each hind paw of rats were obtained from day 1, following the standard evaluation process.^[^ ^34^ ^]^ Score 0: no evidence of erythema and swelling occurred. Score 1: erythema and mild swelling appeared. Score 2: erythema and mild swelling extended from the ankle to the tarsals. Score 3: erythema and moderate swelling extended from the ankle to metatarsal joints. Score 4: erythema and severe swelling encompassed the ankle, paws, and digits or ankylosis of the limb. The final arthritis score of each rat was the sum score of the total four limb scores.

### Micro‐CT Imaging

To evaluate the bone damage, the ankle joints of rats sacrificed at day 29 were fixed in 10% buffered formalin for a week and scanned at 70 kV and 100 µA with the resolution of 20 µm in micro‐CT (ZKKS‐MCT‐Sharp) for 50 min. Then, the dataset was reconstructed using ZKKS‐MicroCT 4.1 workplace to obtain the 3D images of joints and to measure the ratio between bone surface and bone volume (BS/BV).

### Histological Analysis and Immunohistochemical Staining

At study endpoints, rats were euthanized and their hind knee joints were collected for H&E or safranin‐O staining. The image was obtained by microscopy (NIKON ECLIPSE CI, USA). Quantification of cartilage area was carried out by Safranin‐O staining of tissue. For immunohistochemical staining, joint sections were stained with an anti‐HIF‐1*α* antibody. Biotinylated anti‐rabbit IgG was then used as the secondary antibody for chromagen development. Sections were developed using the DAB substrate and then counterstained with haematoxylin. The images were captured by NIKON ECLIPSE TI‐SR. For evaluation of synovial inflammation of the joints, each histopathologic feature was graded by a trained pathologist (SML) using a scoring system as previously described:^[^ ^35^ ^]^ synovial cell lining hyperplasia (0–3); pannus formation (0–3), and inflammatory cellular infiltration (0–3). The synovitis score of each joint was the sum of all the histopathologic feature scores.

### Quantification of Serum Cytokines

Serum samples from the CIA rats were collected on day 28 and concentrations of IL‐10, IL‐6, and TNF‐*α* were quantified with ELISA. Briefly, the whole blood was collected by tail‐cutting and then allowed to clot at room temperature for 30 min. Samples were then centrifuged at 4000 × *g* for 20 min to collect serum from the supernatant. Serum samples were immediately frozen at −20 °C until analysis by using rat IL‐10, rat IL‐6, and rat TNF‐*α* ELISA kits (Dakewei) within 3 days of collection.

### qRT‐PCR Analysis From Collected Synovial Joints

At day 29 post‐adjuvant administration, synovial tissues from knee joints were collected using sterile surgical blades. The obtained tissues were minced with surgical blades and extracted with Super Total RNA Isolation Kit (Foregene). Synthesis of cDNA and qRT‐PCR was performed as previously described for in vitro analysis.

### Hemolysis Assay

2% red blood cells suspension with normal saline (negative control), deionized water (positive control), and MnO_2_‐motors with different concentrations (2.5, 5, 10, and 20 µg mL^−1^) were shaken for 2 h at 37 °C. All samples were centrifuged, and the supernatants was analyzed at 570 nm by a microplate reader.

### Statistics and Data Analysis

PRISM software 8.0 (Graph Pad Software) was used. The mean ± SD were determined for all treatment groups. Two‐sample comparisons were performed by Student's *t* test and multiple comparisons were conducted by a one‐way analysis of variance (ANOVA) followed by post hoc testing. *p* < 0.05 was considered representative of a statistically significant difference between two groups.

## Conflict of Interest

The authors declare no conflict of interest.

## Author Contributions

*Conceived and designed the experiments*: C.X. and Y.T. The manuscript was written through the contributions of all authors. All authors have given approval to the final version of the manuscript.

## Supporting information

Supporting Information

Supplemental Movie 1

Supplemental Movie 2

Supplemental Movie 3

## Data Availability

The data that support the findings of this study are available from the corresponding author upon reasonable request.
