# Supplementary material for: Arthritic Microenvironment Actuated Nanomotors for Active Rheumatoid Arthritis Therapy
Source: Adv Sci (Weinh). 2022 Nov 14;10(4):2204881. doi: 10.1002/advs.202204881 (PMC9896045; doi:10.1002/advs.202204881)
Supplement: Supplementary file 1 — Supporting Information [file ADVS-10-2204881-s003.pdf]

ADVANCED SCIENCE
Open Access

Supporting Information

for *Adv. Sci.*, DOI 10.1002/advs.202204881

Arthritic Microenvironment Actuated Nanomotors for Active Rheumatoid Arthritis Therapy

Cong Xu, Yuejun Jiang, Hong Wang, Yuxin Zhang, Yicheng Ye, Hanfeng Qin, Junbin Gao, Qing Dan, Lingli Du, Lu Liu, Fei Peng, Yingjia Li* and Yingfeng Tu**

Supporting Information

Arthritic Microenvironment Actuated Nanomotors for Active Rheumatoid Arthritis Therapy

Cong Xu, Yuejun Jiang, Hong Wang, Yuxin Zhang, Yicheng Ye, Hanfeng Qin, Junbin Gao, Qing Dan, Lingli Du, Lu Liu, Fei Peng, Yingjia Li* and Yingfeng Tu**

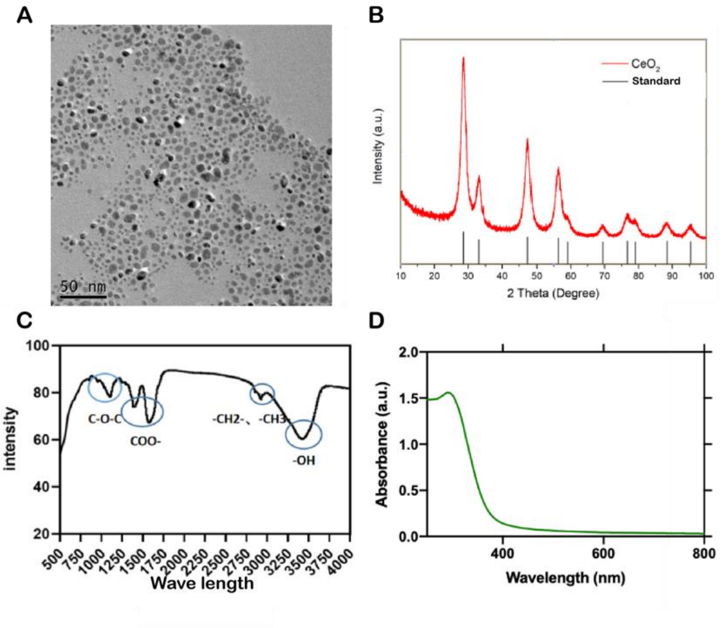

Figure S1. Characterization of ceria NPs. (A) TEM image of ceria NPs. (B) X-ray powder diffraction of ceria NPs. (C) infrared spectrum of ceria NPs. (D) UV-vis spectra of ceria NPs.

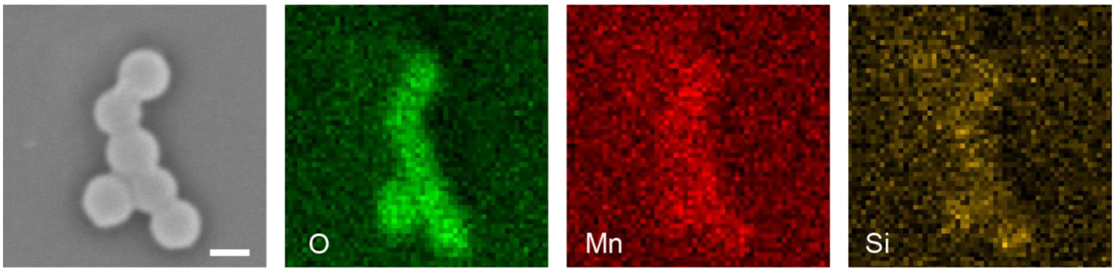

Figure S2. SEM image of MnO_2-motor, and the corresponding element mapping of O, Mn and Si respectively. Scale bar=200 μm.

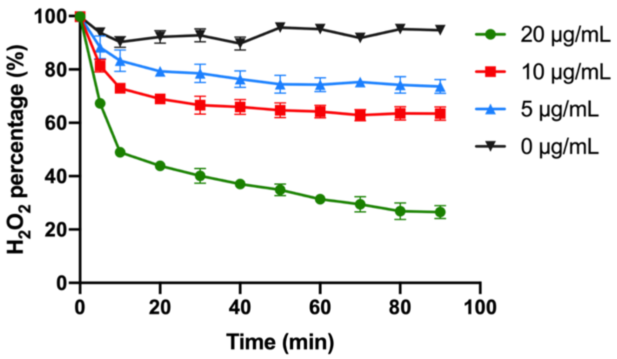

Figure S3. H_2O_2 degradation curves in the presence of MnO_2-motors under 2.5 mM H_2O_2 solution(n = 4; mean ± SD).

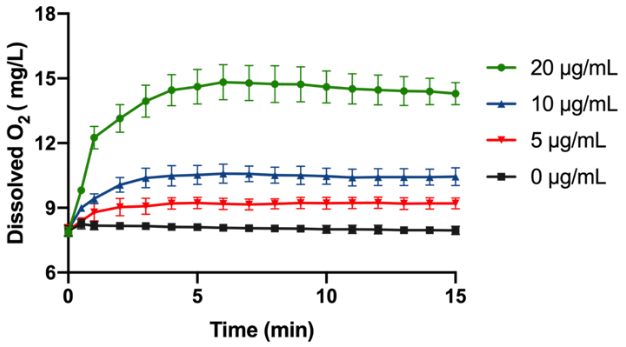

Figure S4. O_2 generation of MnO_2-motors with different concentrations in 100 mM H_2O_2 solution (n = 4; mean ± SD).

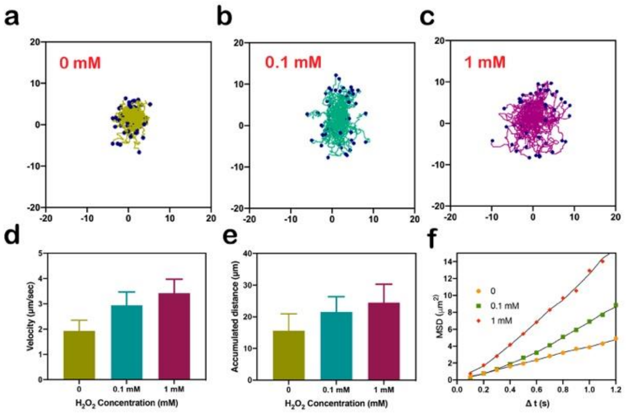

Figure S5. (a-c) Enhanced diffusion of MnO_2-motors under SSF at different H_2O_2 concentrations. (d) Velocity of MnO_2-motors. (e) Accumulated distance of MnO_2-motors. (f) MSD curve of MnO_2-motors. The motion of nanomotors was analyzed with ImageJ for 10 s (n = 52).

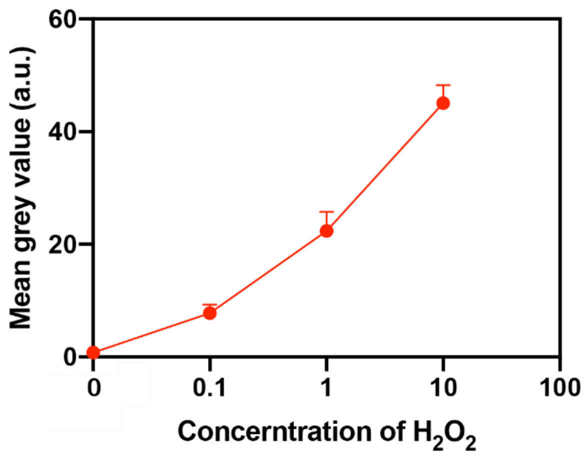

Figure S6. The change of mean gray value of the *in vitro* images measured by Image J. (n = 3; mean ± SD)

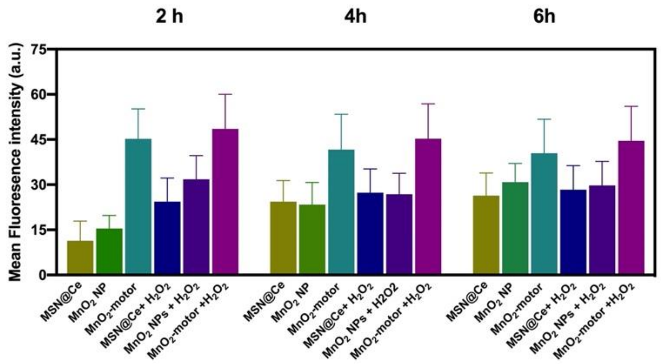

Figure S7. Mean fluorescence intensity of RAW264.7 cells after incubating with MnO_2-motors (n = 3; mean ± SD).

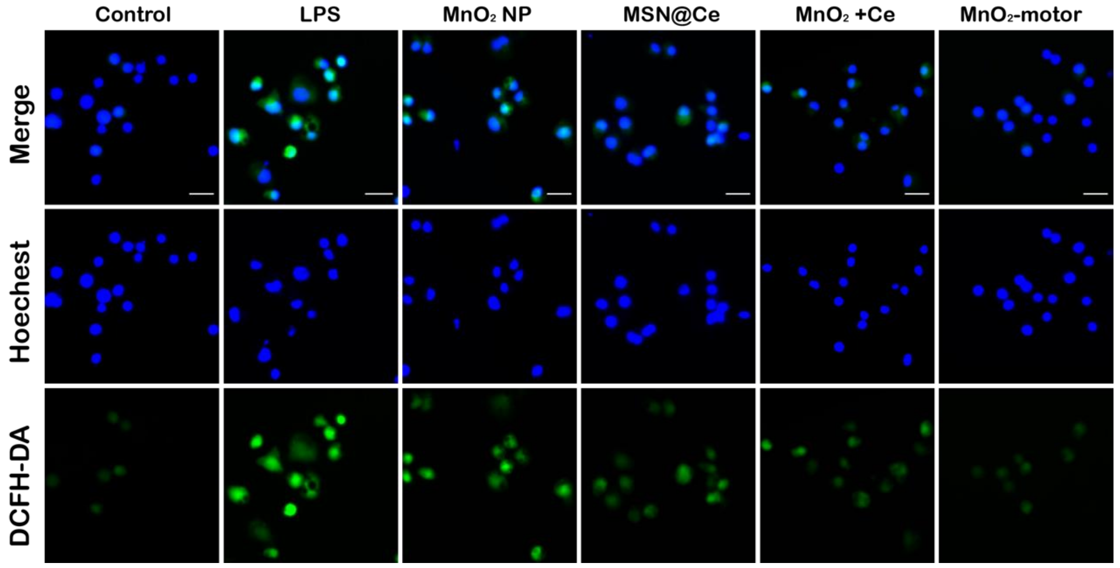

Figure S8. Inverted fluorescence microscopy images of ROS in RAW264.7 cells probed by DCFH-DA. Scale bar=50 µm.

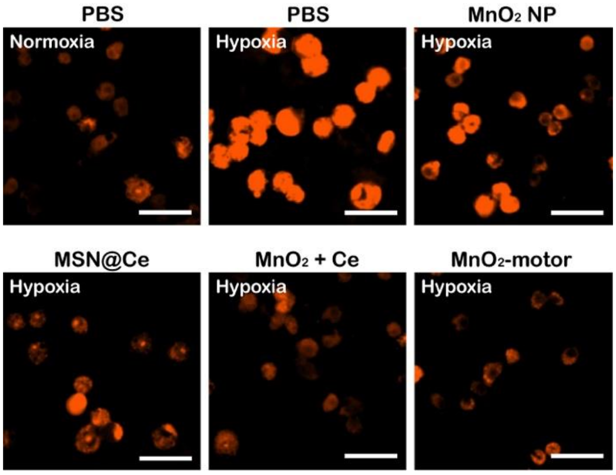

Figure S9. Inverted fluorescence microscopy images of O_2 indicator in RAW264.7 cells. Scale bar=50 μm.

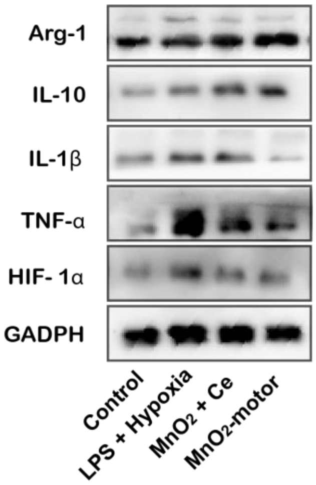

Figure S10. Protein expression of HIF-1α, M1 (IL-1β and TNF-α), and M2 (Arg-1 and IL-10) macrophage markers in RAW264.7 cells after different treatments, as evaluated by Western blot analysis.

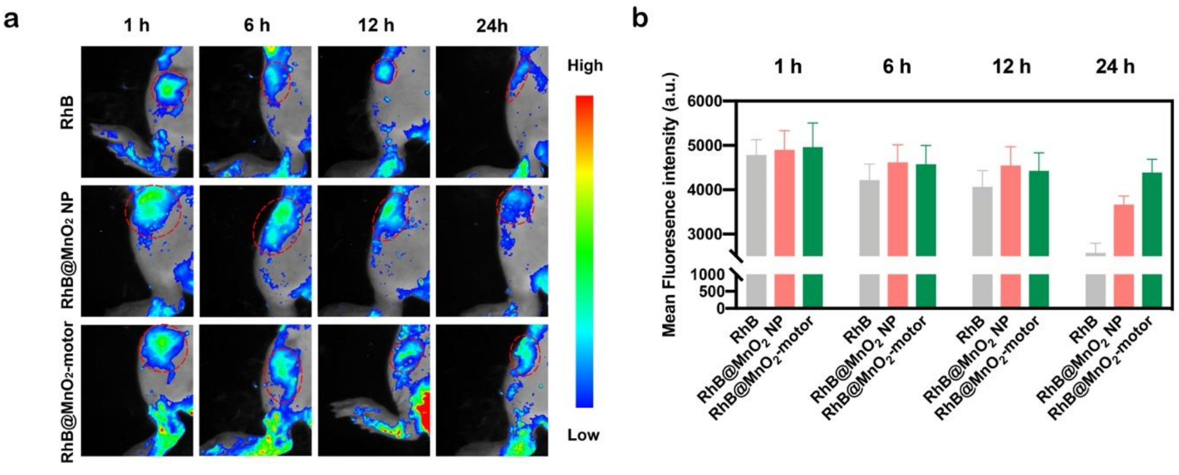

Figure S11. (a) Distribution of Rhodamin B-labeled MnO_2-motors in joints compared with free Rhodamin B and Rhodamin B-labeled MnO_2 NP. (b) Fluorescence intensity of MnO_2-motors in joints.

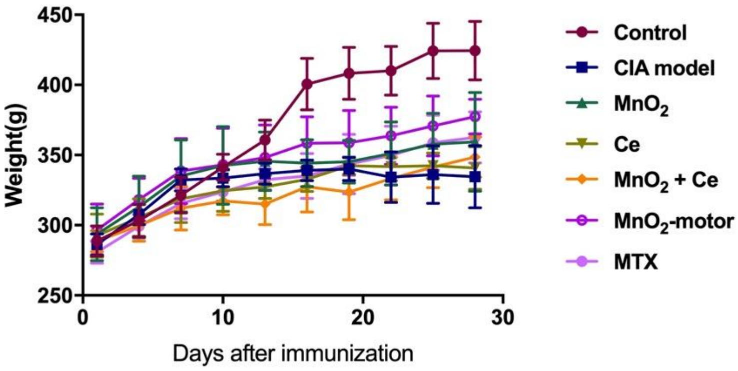

Figure S12. Weight changes of rats after different treatments (n = 5; mean ± SD)

.

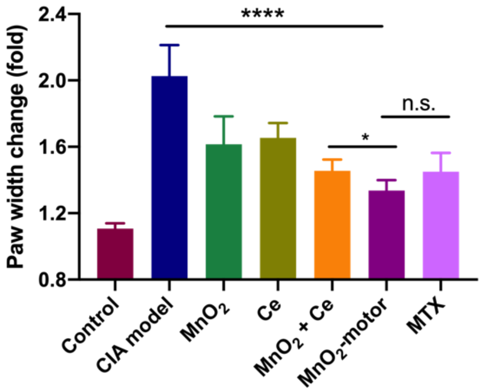

Figure S13. Paw width change of rats after different treatments (n = 5; mean ± SD) (one-ANOVA test).

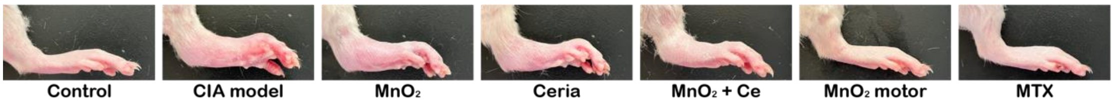

Figure S14. Representative photographs of hind legs taken on the 28th day.

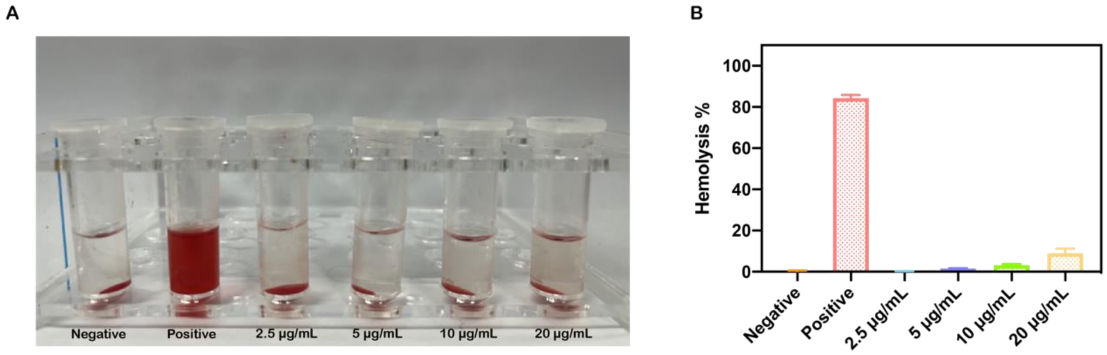

Figure S15. Hemolysis assay of MnO_2-motor. (A) Photographs and (B) hemolysis assay of negative, positive and MnO_2-motor with different concentrations (n = 3; mean ± SD).

Movie S1. Motion of MnO_2-motors in H_2O_2 solution with different concentrations. (MP4)

Movie S2. In vitro O_2 generation of MnO_2-motors under H_2O_2 solution with different concentrations. (MP4)

Movie S3. Ultrasound detection of a CIA joint as compared with a normal joint. (MP4)
